# Metaboloepigenetic Regulation of Pluripotent Stem Cells

**DOI:** 10.1155/2016/1816525

**Published:** 2015-12-29

**Authors:** Alexandra J. Harvey, Joy Rathjen, David K. Gardner

**Affiliations:** ^1^Stem Cells Australia, Parkville, VIC 3010, Australia; ^2^School of BioSciences, The University of Melbourne, Parkville, VIC 3010, Australia; ^3^School of Medicine, University of Tasmania, Hobart, TAS 7000, Australia

## Abstract

The differentiation of pluripotent stem cells is associated with extensive changes in metabolism, as well as widespread remodeling of the epigenetic landscape. Epigenetic regulation is essential for the modulation of differentiation, being responsible for cell type specific gene expression patterns through the modification of DNA and histones, thereby establishing cell identity. Each cell type has its own idiosyncratic pattern regarding the use of specific metabolic pathways. Rather than simply being perceived as a means of generating ATP and building blocks for cell growth and division, cellular metabolism can directly influence cellular regulation and the epigenome. Consequently, the significance of nutrients and metabolites as regulators of differentiation is central to understanding how cells interact with their immediate environment. This review serves to integrate studies on pluripotent stem cell metabolism, and the regulation of DNA methylation and acetylation and identifies areas in which current knowledge is limited.

## 1. Introduction

Resurgence in metabolic research has revealed metabolism to be at the heart of cell-sensing mechanisms. Not only does metabolism provide ATP to maintain homeostasis and cell replication and intermediates that form the basic building blocks for cell proliferation, but also metabolic processes and products can modulate signalling pathways, transcription factor activity, and gene expression. Metabolites can induce long-term changes to the cell through the regulation of the epigenome, a phenomenon referred to as metaboloepigenetics. Every cell type has a unique metabolic phenotype and a unique epigenetic profile, reflecting their cellular niche and function. It is hypothesized that not only does the pattern of metabolism observed in different cell types serve to fulfil that cell's specific functions, but also metabolism is involved in establishing the epigenome of the cell during development. This implies that the intra- and extracellular metabolic environment, in which cells reside, either* in vivo* or* in vitro* can have a profound effect on cellular phenotype. Further, the ability of cells themselves to modify their own environment in order to facilitate their function warrants consideration.

The pluripotent epigenome must maintain transcription of pluripotency-related genes, while being poised for rapid, lineage-specific gene activation upon differentiation [1–3]. Concomitantly, cells constantly modulate their metabolic state in response to extracellular signals, including nutrient availability [4]. Significant changes in metabolism accompany the transition from the early embryo through differentiation [5, 6]. The availability and activity of metabolic cofactors and enzyme substrates, generated through cellular metabolism, can impact the regulation of transcription through modulation of epigenetic processes, including histone methylation and acetylation. Metabolism is consequently emerging as a central player in the regulation of epigenetics and gene expression.

Here we review recent advances in our understanding of the roles of metabolites and cofactors in modulating the pluripotent stem cell epigenome. We discuss how stem cell metabolism and chromatin modifications are interconnected, how their interactions can impact stem cell state and differentiation, how culture conditions have the potential to induce (erase/generate) epigenetic marks, how these processes could significantly impact the utility of cells, and the potential for metabolic alterations to induce epigenetic deregulation. We refer the reader to existing reviews on mitochondrial characteristics of pluripotent stem cells [7–9].

## 2. Defining Pluripotent Stem Cell States

In the embryo and in culture, pluripotent cells have been shown to comprise a lineage of temporally distinct cell states (reviewed in [10]). Pluripotent stem cells, either embryonic (derived from the inner cell mass (ICM) of the blastocyst stage preimplantation embryo; ES cells) or reprogrammed from a somatic cell to an embryonic stem cell-like state (induced pluripotent stem cells; iPS cells) are defined by their ability to self-renew (to proliferate indefinitely) and by pluripotency, as shown by the ability to act as a founder cell population for all the cells of the embryo and adult. These properties underpin the potential use of these cells as a source of clinically relevant cells for therapeutics and drug discovery. Many studies have focused on defining the molecular properties of ES cells but only recently have we begun to investigate the physiology and metabolism of these cells.

Mouse and human ES cells differ in their growth factor requirements* in vitro*, a consequence of their origins from different developmental stages. Mouse ES cells isolated from the ICM are reliant on leukemia inhibitory factor (LIF) for ongoing propagation, while also requiring serum [11]. Alternatively, mouse ES cells can be isolated in medium supplemented with inhibitors of Mek/Erk and GSK3 activation [12]. Human ES cells are derived from a later stage pluripotent cell population, more similar to postimplantation epiblast [13], and are dependent on activin/nodal and fibroblast growth factor (FGF) signaling for self-renewal and pluripotency [14–16]. The tissue of origin and gene expression profile of human ES cells suggest that they are representative of a later stage pluripotent cell state. Pluripotent cells have been isolated from the postimplantation epiblast or primitive ectoderm of the mouse. Like human ES cells, epiblast stem cells (EpiSC) require FGF and activin A for self-renewal and pluripotency [17–19]. In culture these cells adopt the phenotype of the anterior primitive ectoderm from the late gastrula stage embryo [17].

Mouse ES cells can be cultured with inhibitors of Fgf, Mek/Erk, and Gsk3 to form a naïve ES cell state, representative of the early inner cell mass [20–22]. Inclusion of the GSK3 inhibitor minimizes the negative regulation of biosynthetic pathways [12], thereby modulating proliferative capacity. Alternatively, mouse ES cells can be cultured in medium supplemented with the amino acid l-proline to form early primitive ectoderm-like (EPL) cells that represent a stage of pluripotency intermediate to ES cells and EpiSC [23].

Each population, isolated or cultured, represents a stem cell state within the continuum of the pluripotent lineage.

## 3. Framework of Pluripotent Stem Cell Metabolism

**Figure 1:**
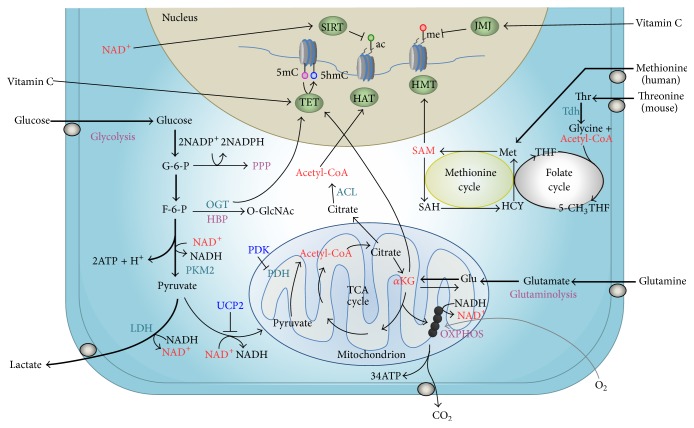
Metabolic regulation of the pluripotent epigenetic landscape. Pluripotent stem cells are characterized by spherical, electron-poor mitochondria, which contain few cristae. These cells rely heavily on glycolysis for ATP generation (thick black arrows), resulting in significant lactate production through the conversion of pyruvate to lactate by lactate dehydrogenase (LDH). Consequently, oxidative phosphorylation (OXPHOS) contributes minimally to total ATP. Glucose metabolized by the pentose phosphate pathway (PPP) generates the ribose moieties required for DNA and RNA synthesis and the NADPH required for the biosynthesis of lipids and other complex molecules. Intermediate metabolites generated via metabolic pathways act as cofactors for epigenetic modifying enzymes. Threonine and methionine metabolism is required for S-adenosyl methionine (SAM) generation via the folate and SAM cycles in mouse and human pluripotent stem cells, respectively [60, 61]. SAM acts as a methyl donor for histone methyltransferases (HMT) as well as DNA methyltransferases. Demethylation of SAM yields S-adenosylhomocysteine (SAH), which is, in turn, hydrolyzed to homocysteine. Transfer of a methyl group to homocysteine from the folate pathway regenerates methionine. Acetyl coenzyme A (Acetyl-CoA) acts as a cofactor for histone acetyltransferases (HAT). Acetyl-CoA, generated from glucose derived pyruvate, modulates human pluripotent stem cell histone acetylation [76], although pyruvate dehydrogenase kinase (PDK) activity may limit the conversion of pyruvate to acetyl-CoA [79]. Similarly, uncoupling protein 2 (UCP2) functions to shunt pyruvate away from the tricarboxylic acid (TCA) cycle, facilitating lactate production [79]. Acetyl-CoA can also be generated from threonine catabolism [62]. Alpha-ketoglutarate (*α*KG) [65] or supplementation with vitamin C in culture [122] reduces histone and DNA methylation in human pluripotent stem cells, respectively. These metabolites modulate histone and DNA demethylation reactions catalyzed by Jumonji (JMJ) and Ten-Eleven Translocation (TET) demethylases, respectively [101]. The hexosamine biosynthetic pathway (HBP) is an alternative route of glucose utilization that generates the coenzyme UDP-GlcNAc, which together with O-linked N-acetylglucosamine transferase (OGT) leads to histone O-GlcNAcylation [82]. Flux through glycolysis and oxidative phosphorylation determines the NAD^+^ : NADH ratio, known to regulate the activity of the NAD^+^-dependent histone deacetylases sirtuins (SIRT; [90]). In addition, a proline-dependent mechanism of epigenetic regulation has been reported in pluripotent stem cells [74]; however, it is unclear how the metabolism of proline interacts with these pathways. Metabolic regulators of chromatin-modifying enzymes are highlighted in red.

Pluripotent stem cells and the pluripotent cells of the ICM from which they are derived exhibit a metabolism characterized by high levels of glucose consumption, combined with the production of lactate (reviewed by [9, 24]; Figure 1). This pattern of metabolism is maintained in conditions of oxygen sufficiency, distinguishing it from anaerobic glycolysis, and has therefore been termed aerobic glycolysis. Warburg [25] first described aerobic glycolysis in cancer cells in culture, which produce large amounts of lactate even in the presence of sufficient oxygen for the complete oxidation of glucose (the Warburg effect). Initially it was thought that aerobic glycolysis was specific to cancers, but it has since been shown that this metabolic trait occurs in other proliferating cells types such as lymphocytes [26]. Gardner [27, 28] identified similarities between cancers and blastocysts and the significance of the Warburg effect for the development of the late stage embryo.

What are the cellular advantages of aerobic glycolysis to pluripotent stem cells? Glucose is typically considered in its capacity as an energy source. Pluripotent cells are highly proliferative, with reports of cell cycle rates reducing to as little as 5–7 hours in the developing embryo [29]. To maintain this growth rate these cells will have to generate building blocks for proteins, nucleic acids, lipids, and carbohydrates. The glycolytic metabolism of glucose may not generate as much ATP as oxidative phosphorylation per mol of glucose, but it can readily create equal amounts of ATP by an increased flux of glucose. Further, enhanced glycolytic rate plays a significant role in the generation of metabolic intermediates for biosynthesis. The carbon in glucose is utilized in the synthesis of triacylglycerols and phospholipids. Glucose is precursor for complex sugars of mucopolysaccharides and glycoproteins [27, 28]. Metabolism of glucose through the pentose phosphate pathway (PPP) generates ribose moieties required for DNA and RNA synthesis and the NADPH required for the biosynthesis of lipids and other complex molecules [30, 31]. Aerobic glycolysis is therefore the mechanism that ensures sufficient carbon flux through biosynthetic pathways in rapidly dividing cells [27, 30, 32, 33].

The preferential metabolism of glucose through glycolysis reduces the use of oxidative metabolism in pluripotent cells, with a concomitant decrease in the formation of reactive oxygen species (ROS) associated with oxidative phosphorylation [34]. From a developmental perspective, this reduces oxidative stress within the cell, thereby reducing the risk of DNA damage. Given that pluripotent cells* in vivo* and* in vitro* act as founders for all cell types of the embryo and adult, a metabolism that promotes genetic stability would represent an evolutionary adaptation for successful and faithful propagation.

## 4. Key Metabolites Define the* In Vivo* Pluripotent Stem Cell Niche

Maintenance of pluripotency relies on a balance of complex cellular and acellular signals within the surrounding microenvironment. High levels of aerobic glycolysis in pluripotent cells form a localized area or niche, characterized by relatively high concentrations of lactate and low extracellular pH surrounding the blastocyst (and potentially around cell colonies in culture). The blastocyst uses this microenvironment to facilitate the implantation process [24]. This environment assists in extracellular matrix degradation, angiogenesis, and immune-modulation of the mother at the implantation site. Lactate, as it would appear, is a very important signalling molecule that elicits numerous effects in the cell of origin and surrounding tissues. Some of these effects could be modulated through lactate-responsive transcription factors. Many cancers appear to recreate an embryonic-like phenotype and coopt embryonic pathways. Cancers, like blastocysts, generate a microenvironment characterized by high lactate and reduced external pH, created through aerobic glycolysis, to facilitate tissue invasion, angiogenesis, and immunomodulation. The role of such a microenvironment in* in vitro* stem cell culture has not been considered, though it is likely to have a profound effect on pluripotent stem cells and those cells surrounding them.

Low oxygen is a characteristic of the stem cell niche* in vivo*, where the oxygen concentration within the reproductive tract approximates 2–8% [35, 36]. Within the oviduct, early embryo development takes place in an oxygen concentration from 5 to 8.5% (35–60 mm Hg) in the rabbit, hamster, and rhesus monkey [35]. Around the time of compaction, coinciding with the first lineage specification event, the embryo traverses into the uterus, which has a lower oxygen concentration of 1.5–2% oxygen in the rhesus monkey, 3.5% in the rabbit, 5% in the hamster [35], and 4% in the rat [36]. Decreased oxygen in the uterus is particularly evident at the time of implantation, when in rabbits and hamsters a decrease from 5.3% to 3.5% oxygen is seen [35]. Therefore, embryos appear to encounter a decreasing oxygen concentration gradient as they progress from the oviduct to the uterus. Furthermore, during the time of early implantation, hypoxia and even anoxic conditions confront the invading trophectoderm (reviewed by [37]). At concentrations of 7% oxygen and lower the activation of hypoxia-inducible factors (HIFs; [38], reviewed by [39]) occurs within cells. HIFs modulate cellular homeostasis through the regulation of glucose metabolism, pH, angiogenesis, and iron metabolism, supporting a high rate of glycolysis.

## 5. Regulation of Epigenetic Modifiers by Metabolic Cofactors

Cell state transitions are characterized by global changes in the epigenetic landscape [40, 41]. As differentiation proceeds, epigenetic modifications progressively restrict gene expression, silencing pluripotency genes and activating lineage-specific genes [42]. Underlying pluripotency, ES cells are characterized by an open and highly dynamic chromatin landscape (reviewed by [43]). Progression of the pluripotent lineage and early events in differentiation are accompanied by changes in the genomic architecture. This is evidenced in changes in mean replication timing (MRT) at loci across the genome. Changes in MRT provide evidence of changes in the genomic organization that underpin the establishment of cell identity [40]. Large changes in MRT accompany the global genome reorganization, also known as autosomal lyonization, and occur as EPL cells differentiate to cells representative of a later primitive ectoderm (EpiSC and EBM6; [44]) and the germ lineage progenitors. Coincident with autosomal lyonization are changes in the nuclear architecture and the formation and accumulation of late-replicating heterochromatin at the nuclear periphery [44].

Stable modifications to DNA are catalyzed by DNA methyltransferases (DNMTs). In general, DNA methylation can modify chromatin architecture and prevent transcription factor binding within gene promoters, restricting gene expression. Methylation of lysine and arginine residues within histones H3 and H4 is catalyzed by residue-specific methyltransferases (HMT) and can be associated with either transcriptional repression or activation. Establishment of methylation patterns such as H3K4 di- or trimethylation (H3K4me2/me3) and H3K27 trimethylation (H3K27me3) are generally associated with transcriptional activation, while H3K9me2/3 and H3K27me2/3 are associated with transcriptional repression. S-Adenosyl methionine (SAM) acts as the primary methyl donor for DNA and histone methylation, generated through one-carbon metabolism. This pathway integrates the folate and methionine cycles (Figure 1), the latter having metabolic inputs from methionine, serine, and glycine metabolism. ES cells are characterized by elevated global transcriptional activity [45]. Repressive marks, such as H3K9me3, are low in ES cells compared with differentiated cells [46]. Bivalent methylation, marked by a combination of active H3K4me3 and repressive H3K27me3 at a subset of developmental regulators, has been proposed to establish a primed epigenetic state, ready for activation prior to ES cell differentiation [2] and to safeguard differentiation [3].

DNA demethylation takes place either passively or actively. Passive demethylation occurs with DNA replication in the absence of maintenance methyltransferases. The process of active demethylation is catalyzed by Ten-Eleven Translocation (TET) dioxygenases, responsible for the conversion of 5-methylcytosine (5mC) to 5-hydroxymethylcytosine (5hmC) [47–49]. TET activity is dynamically regulated by alpha-ketoglutarate (*α*KG), a product of the TCA cycle, and succinate [50]. Tet1 and Tet2 are highly expressed in mouse ES cells [51], with Tet1 also enriched in the inner cell mass of mouse blastocysts [51]. High 5hmC levels are present in mouse ES cells and decrease significantly after differentiation [49, 52]. In a similar manner, Jumonji demethylases are regulated by *α*KG [53]. Jmjd1a and Jmjd2c knockdown leads to mouse ES cell differentiation, regulating pluripotent gene expression [54].

Histone acetylation participates in multiple chromatin-dependent processes, including gene regulation, DNA replication, and DNA damage repair. Acetylation is generally associated with a more open chromatin configuration, permissive to transcription, while deacetylation is associated with condensed, compact chromatin leading to transcriptional repression. Acetylation is catalyzed by histone acetyltransferases (HATs), which transfer an acetyl group from acetyl coenzyme A (acetyl-CoA) to lysine residues, with the concomitant production of CoA. Cellular acetyl-CoA levels fluctuate in response to various physiological cues, including nutrient availability and metabolic activity. A major source of acetyl-CoA in cells is the conversion of citrate via ATP citrate lyase (ACL). siRNA-mediated silencing of ACL significantly decreases histone H2B, H3, and H4 acetylation in HCT116 colorectal cancer cells [55]. Glycolysis plausibly has a significant role in modulating acetyl-CoA levels, and glucose availability can affect histone acetylation in an ACL-dependent manner [55].

Histone deacetylation is catalyzed by NAD^+^-independent or NAD^+^-dependent deacetylases (HDACs). Class I and II HDACs are dependent on zinc, while the activities of the sirtuin family of histone deacetylases (class III) are reliant on NAD^+^ for their catalytic activity. Sirtuins (SIRTs) act as sensors of environmental stimuli and deacetylate histone and nonhistone substrates. In addition, they have roles in the regulation of a number of metabolite pathways, including glycolysis, the TCA cycle and fatty acid oxidation, telomere maintenance, tolerance to oxidative stress, and DNA repair. High rates of glycolysis establish a high NADH/NAD^+^ ratio, which downregulates sirtuin activity. Studies of pluripotent stem cell histone acetylation have largely focused on the regulation of deacetylation by class I and II HDACs and their inhibitors, in the context of differentiation. A genome-wide reduction of H3K9 acetylation (H3K9ac) is required for mouse and human ES cell differentiation [56, 57]. Partial inhibition of ES cell HDAC activity has been shown to promote ES cell self-renewal [58, 59]. Therefore, acetylation maintains a highly dynamic configuration permissive to transcriptional activation.

## 6. Linking Pluripotent Stem Cell Metabolism with Epigenetics and Cell State

### 6.1. The Role of Essential Amino Acids in Modulating Pluripotent Stem Cell Methylation

Pivotal studies in mouse ES cells highlight the role of specific amino acids in pluripotent cell regulation. Depletion of individual amino acids from ES cell culture identified threonine as a critical regulator of pluripotency. Threonine catabolism supports mouse ES cell self-renewal, while elimination of threonine from culture medium results in slowed proliferation and increased differentiation [60, 61]. Threonine catabolism contributes to cellular glycine and acetyl-CoA levels, the former being required for SAM synthesis through the SAM cycle (Figure 1). Depletion of threonine from the culture medium or knockdown of threonine dehydrogenase (Tdh) in mouse ES cells decreased SAM accumulation [60] and altered differentiation potential [62]. Analysis of ^13^C-labelled threonine demonstrated that threonine contributes significantly to the acetyl-CoA pool in mouse ES cells and that glycine derived from ^13^C-Thr contributed to SAM synthesis. Removal of threonine leads to the loss of methylated histone H3. Threonine reduction, not sufficient to induce cell death, was accompanied by a decrease in H3K4me3 [62], suggestive of a more repressive epigenetic landscape. Loss of H3K4me3 could be rescued by supplementation with threonine or with glycine and pyruvate, associated with an increase in the SAM/SAH ratio [62].

Human ES cells also require SAM but generated through an alternative metabolic pathway, a consequence of their lack of a functional TDH [63]. Systematic elimination of amino acids from culture medium identified methionine as a critical amino acid. Methionine deprivation resulted in a reduction in cell number within 5 hours, attributable to increased cell death, and was associated with a reduction in SAM levels and NANOG expression [64]. Knockdown of the methionine adenosyltransferases, MAT2A and MAT2B, that catalyze the conversion of methionine to SAM similarly decreased cell numbers after 48 h, suggesting that SAM, rather than methionine, was essential for cell survival. The early reduction in cell numbers on methionine depletion is reversible but later impacts on cell proliferation following prolonged methionine deprivation are not [64]. We interpret this to mean that maintenance of SAM levels is critical for stem cell survival and reductions in the metabolite interfaces with apoptosis machinery. Short (5 h) and long (24 h) term methionine deprivation led to a rapid decrease in H3K4me3, accompanied by a modest reduction in global DNA methylation; the effects of short term deprivation could be abrogated through supplementation with SAM [64].

These approaches identify SAM as a major methyl donor within pluripotent cells and show that reduction of SAM reduces histone methylation. The processes used to reduce SAM within the cells likely reduce levels below physiological ranges and impact on the pools of other important metabolites. Shyh-Chang et al. [62] detected reduced NADH/NAD^+^ and glycine and increased ATP, glucose-6-phosphate, and fructose-6-phosphate, within the first 6 hours of threonine depletion, suggesting that culture media are rapidly depleted of other nutrients, essentially starving cells. This alone may account for the increased cell death observed in both studies, particularly as human ES cells were shown to replenish SAM within 24 hours through recycling of homocysteine [64]. These data suggest that threonine and potentially methionine are critical to maintain metabolic balance and therefore cell survival, within pluripotent stem cells in roles independent of SAM generation. The question remains whether the modulation of SAM concentrations within cells within a physiological range provides a mechanism for the cells to link their metabolome with their epigenome. These data suggest that homocysteine may be important in modulating cell survival. The methionine pathway is reliant on transfer of a methyl group to homocysteine from the folate pathway to regenerate methionine. The ability of glycine, in the presence of pyruvate, to restore cell survival after short term methionine withdrawal implicates the folate pathway in modulating pluripotent cell survival.

Shiraki et al. [64] noted that short term methionine deprivation potentiated subsequent cell differentiation, with more cells exhibiting lineage-specific marker expression on day 4 when induced to differentiate with known differentiation-inducing conditions. It will be important to understand whether methionine deprivation potentiates differentiation by poising cells in a more primed state relative to methionine supplemented conditions or whether methionine deprivation selects a population of cells more receptive to differentiation-inducing conditions.

### 6.2. Glutamine Regulates ES Cell Methylation

Glutamine has been shown to regulate pluripotency and histone methylation. Most proliferating mammalian cells rely on the catabolism of two molecules, glucose and glutamine, to fulfill their energy, carbon, and nitrogen requirements [4]. As expected, naïve and primed cells consume glucose and glutamine, although steady-state levels of TCA cycle intermediates were lower in naïve ES cells [65]. Neither naïve nor primed mouse ES cells were able to proliferate in the absence of glucose [65], demonstrating an absolute requirement for this metabolite. These cell states could, however, be distinguished by their ability to proliferate in the absence of glutamine, with naïve, but not primed, ES cells able to proliferate, albeit at a reduced rate, in glutamine deficient medium [65]. The proliferation of naïve ES cells in glutamine-deficient medium was supported by an increase in glutamate production from glucose, while the addition of precursors of glutamine synthesis to the medium of primed ES cells enabled proliferation in glutamine-depleted medium [65]. This suggests that the transition from naïve to primed ES cells establishes a reliance on glutamine and TCA cycle activity to support proliferation.

Naïve ES cells exhibited an increased *α*KG : succinate ratio (Carey et al. 2015), where elevated *α*KG could impact the epigenome through modulation of Jumonji and TET activity. Following glutamine deprivation, increased H3K9me3, H3K27me3, H3K26me3, and H4K20me3 levels were detected in naïve ES cells, which could be reversed through medium supplementation with cell permeable *α*KG [65]. These results suggest that the high levels of intracellular *α*KG found in naïve ES cells, sustained through glucose-dependent glutamate production, maintain an epigenetic landscape characterized by low levels of histone methylation. As cells progress to the primed state, their metabolic phenotype changes, glucose-dependent glutamate production and intracellular *α*KG levels decrease, and a concomitant increase of histone methylation accumulates, consistent with the higher levels of methylation seen in these ES cells.

### 6.3. l-Proline Metabolism Induces Changes in the Epigenome That Reflect Pluripotent Cell Identity

Pluripotent early primitive ectoderm-like (EPL) cells can be formed from primed ES cells in culture [66]. The loss of ICM- and ES cell-specific marker gene expression, coupled with increased expression of the primitive ectoderm markers [23, 66–68], increased proliferation rate [23], and a restricted ability to form cell populations characteristic of the primitive endoderm lineage [69, 70] show EPL cells to be distinct from ES cells and align them with the embryonic primitive ectoderm. The amino acid l-proline has been shown to induce the differentiation of ES cells to EPL cells [23, 71–73]. l-Proline activity is facilitated by uptake via the amino acid transporter, SNAT2; inhibition of l-proline uptake through SNAT2 prevents EPL cell formation [71]. l-Proline activity is reliant on intracellular l-proline concentration and l-proline metabolism. Inhibition of proline dehydrogenase prevents the formation of EPL cells ([72], Rathjen unpublished), and removal of l-proline from EPL cells leads to the reestablishment of the ES cell phenotype. Suppression of l-proline biosynthesis, and creation of a shortage within the cell, has been hypothesized to safeguard ES cell identity and prevent autoregulation of differentiation [73].

The addition of l-proline to ES cells induces changes to the epigenome and transcriptome. Analysis of the gross genomic organization of ES and EPL cells has shown these cells to be similar, but repeatable changes in MRT do occur with EPL cell formation and these can be used to distinguish the two cell types [40]. It follows that the metabolism of l-proline and the cell identity changes induced by this process are manifest in changes to genome architecture. Analysis of histone methylation patterns in proline-treated cells showed that epigenetic remodelling, in part, regulated changes to the transcriptome. The addition of l-proline increased the methylation of H3 at lysines 9 and 36 and induced a reprogramming of H3K9 and H3K36 methylation status across the genome [74]. Changes in methylation correlated with loci that were regulated by l-proline. The epigenetic changes induced by l-proline were suppressed when ascorbic acid was added to the cells; to date, the method of ascorbic acid action is not known. The availability of l-proline to the ES cell, which represents the balance of l-proline availability and synthesis in the cell and subsequent metabolism, regulates pluripotent cell identity, with low availability enforcing an ES cell state and increased levels of l-proline inducing EPL cell formation [23, 71, 73]. As part of this process l-proline induces changes to the epigenome characteristic of the EPL cell state.

### 6.4. Glucose Regulation of the Pluripotent Stem Cell Epigenetic Landscape

A high glycolytic rate drives citrate synthesis, leading to the production of cytosolic acetyl-CoA. In turn, acetyl-CoA can act as a cofactor for histone acetylation [75]. Moussaieff et al. [76] have shown that acetyl-CoA levels in human ES cells were twofold higher than those found in their differentiating counterparts. In ES cells, glucose flux through glycolysis was the primary contributor to the acetyl-CoA pool; as cells differentiated the ability to generate acetyl-CoA through this pathway was lost [76]. Acetyl-CoA levels reduce significantly upon mouse ES cell differentiation, although in these cells this was a result of reduced threonine catabolism [61]. The addition of acetate, a precursor of acetyl-CoA, to differentiating human ES cells delayed cell differentiation [76].

The initiation of human ES cell differentiation led to a loss of H3K9/K27 acetylation (H3K9/H3K27ac), marks that are associated with transcriptional repression, while the addition of acetate to differentiating cells blocked this loss. Inhibition of glycolysis by 2-deoxyglucose, previously associated with differentiation [77], similarly decreased H3K9/H3K27ac, an effect that could be reversed with the addition of acetate [76]. The study by Moussaieff et al. suggests that the generation of acetyl-CoA, from glucose-derived pyruvate, is required to support maintenance of a pluripotent epigenetic landscape. Others have questioned the use of pyruvate by pluripotent stem cell mitochondria [78, 79]. Direct quantification of acetyl-CoA flux from pyruvate is needed to clarify the activity of this pathway.

These data support a link between metabolites, metabolism, and epigenetic regulation (summarized in Table 1), but it is still difficult to define this relationship in pluripotent cells. There remains a need to understand more fully the regulation of pluripotent stem cell metabolism, the relative activity of metabolic pathways, and the impact of nutrient availability on pathway activity. The study by Moussaieff et al. (2015) has identified glucose as a source for acetyl-CoA, correlated acetate availability with the level of histone acetylation in pluripotent stem cells, and differentiated derivatives. Coincident with changes in metabolism with differentiation [76], lower levels of H3K9ac have been observed following the initiation of differentiation compared with ES cells [56, 80], along with other global changes in the epigenetic landscape [43, 44]. Conceivably, modulation of the metabolic intermediate pool may serve to facilitate these dynamics and the widespread nature of epigenetic change with the initiation of differentiation.

### 6.5. Other Potential Pathways Modifying Epigenetic Regulator Activity

Pluripotent cells in the embryo and in culture are poised to undergo the most extensive epigenetic regulation event that will occur within the organism's lifespan or during ES cell differentiation, respectively. Not surprisingly, within these cells a number of metabolic pathways appear to be poised to change activity, potentially to provide or limit the pools of modification donors, such as SAM and acetyl-CoA, and to respond to the processes with metabolic regulatory cues. Our knowledge of the metabolic positioning of a pluripotent cell is, however, remarkably sparse. It is highly likely that similar roles for other metabolic pathways in pluripotency, cell state transitions, and lineage specification will be shown. Pathways that are still to be examined include biosynthetic pathways, like the hexosamine biosynthesis pathway and many amino acid synthetic pathways, nutrient sensing mechanisms, and metabolic regulatory mechanisms, such as the sirtuin family of histone deacetylases.

The O-GlcNAc transferase (Ogt) is essential for ES cell viability and loss of the gene disrupts embryogenesis [81] and ES cell self-renewal [82]. More recently, Ogt has been shown to associate preferentially with transcriptional start sites of a number of genes in ES cells and regulate gene expression of genes involved in metabolic and signaling pathways. Ogt associates with Tet1 in a complex in these cells, and Tet1 promotes DNA binding of Ogt [83]. It is tempting to speculate that Ogt is affecting gene regulation in association with Tet1 and through localized epigenetic modifications in gene promoters. The activity of Ogt would appear to be pleiotropic within the cell and alternative mechanisms of gene activation and repression may account for Ogt activity. Further, the colocalization of Ogt, Tet1, and H3K4me3 at hypomethylated, CpG-rich gene promoters may act to maintain these areas free of methylation and modulate the epigenome to maintain the pluripotent state.

It is known that SIRT1, a member of the sirtuin histone deacetylase family, is expressed in the preimplantation embryo and in ES cells [84–86]. Inhibition of SIRTs in the embryo negatively impacts blastocyst development and increases ROS production [87, 88]. In pluripotent cells, SIRT1 levels are downregulated as the cells commence differentiation [84, 89]. These proteins have been shown to play key roles in linking the metabolome with the epigenome by acting as sensors of environmental stimuli, regulating a number of metabolic pathways, and modulating the acetylation of histone and nonhistone substrates (reviewed by [90]). It remains to be determined if any of the roles SIRT1 plays in the early embryo and pluripotent cells are attributable to SIRT1-mediated modulation of histone acetylation and the epigenome.

## 7. Replicating the* In Vivo* Environment and Cell States* In Vitro*

Within the niche the metabolism of pluripotent cells is finely balanced, configured to supply the metabolites required for growth and for maintenance of DNA, and yet poised to respond to the challenges that will be placed on the cell as it differentiates and specifically the requirements of the massive DNA remodeling that accompanies the early events of differentiation. Optimization of* in vitro* culture conditions for pluripotent cells has largely centered on propagating cells that are pluripotent, differentiation competent, and grossly karyotypically normal. It has been assumed that* in vitro* culture conditions, based on commonly used tissue culture media and developed with a focus on growth factor regulation, will sustain the metabolism of the cell. It is known that common culture media are not suitable for embryo culture and significant increases in embryo viability have been achieved by developing media that recapitulate the physiological environment [91]. Our greater understanding of how metabolites can impact not only cell physiology, but also epigenetics, raises questions about the impact of medium metabolite concentrations on the pluripotent epigenome and on cell state.

The composition of the culture medium can significantly influence metabolic pathway use in pluripotent cells, and variations in culture conditions could underlie much of the variability that exists in studies elucidating pluripotent metabolism. We have shown that serum supplementation and supplementation with serum replacer of human ES cell culture changed the production and consumption rates of amino acids and increased glucose uptake by the cells [92]. In contrast to others, however, we were unable to show alterations of metabolism on the induction of differentiation, a difference we ascribe to the maintenance of base medium composition throughout differentiation in our experiments [92]. In a similar way, metabolic differences between naïve and primed ES cells may merely reflect base medium variability. Zhou et al. (2012) attributed metabolic change with cell state without recognizing the potential influence of changing base media [93]. In contrast, Carey et al. (2015) showed that glutamine independence could be established in primed cells independent of base media. Clearly there are metabolic differences between cell states; however it is difficult to distinguish true metabolic differences when base medium composition is not maintained.

A number of protocols for establishing naïve pluripotency have recently been developed for human cells [94–97]. Each of these methodologies used distinct culture conditions that will establish inherent differences in underlying metabolite use. The consequence of these changes in metabolism may not impact pluripotency, differentiation capacity, or gross karyotype but may result in significant alterations to the epigenome. Epigenetic codes will likely be perpetuated during self-renewal and differentiation, potentially influencing future cell events. We have advocated the need to understand the interplay between metabolism and the culture medium to enable true optimization of development in the embryo [98, 99] and in pluripotent stem cells [9].

Abnormalities in cellular metabolism have been linked with alterations in the epigenetic landscape, contributing to numerous diseases including cancer [100, 101]. It will, therefore, be important to establish the metabolic mechanisms regulating pluripotent stem cell epigenetics that underlie pluripotency and differentiation and examine the impact of metabolic perturbations on epigenetic control to ensure these cells and their differentiated derivatives exhibit a normal physiology and are not predisposed to disease states.

### 7.1. Oxygen: The Forgotten Metabolite

The majority of all tissue culture, including the majority of pluripotent cell culture, is performed in the presence of atmospheric oxygen (~20%). The* in vivo* environment in which prevascularisation embryos develop constitutes a relatively low oxygen environment (2–8%). Departure from a physiological oxygen concentration in culture significantly impacts preimplantation embryo development. While embryos are capable of developing under 20% oxygen, this has been associated with increased DNA fragmentation [102–104], altered genomic [105, 106] and proteomic profiles [107], and perturbed metabolic activity [91, 108]. The changes induced by atmospheric oxygen are not consistent with the viability of the blastocyst [99]. Despite the embryonic requirement for physiological oxygen and the negative impact of atmospheric oxygen on embryo viability, cell derivatives of the embryo, including pluripotent cells, are routinely cultured and characterized in 20% oxygen.

We have documented oxygen-dependent changes in pluripotent cell metabolism that occur in the absence of overt changes in standard measures of self-renewal in human ES cells [109]. Others have described similar changes in metabolite use in response to oxygen [110–112], consistent with a conserved cellular response to oxygen availability [39]. Further, the availability of oxygen can significantly impact the pluripotent epigenome. Increased 5′ methylcytosine staining in response to high oxygen has been described in preimplantation embryos [113], with increased expression of SIRT1 and TET1 also reported [114]. Maintenance of blastocyst integrity under low oxygen is likely mediated by HIF2*α* [105]. HIF2*α* has been shown to be responsible for long-term adaptation to hypoxia in human ES cells [115], binding directly to OCT4, NANOG, and SOX2 proximal promoters in human ES cells cultured at low oxygen concentrations [116]. Methylation of the OCT4 hypoxia response element (HRE) in human ES cells is marked by a significant increase in H3K36me3, a marker of transcriptional activation, under 5% oxygen conditions compared with atmospheric oxygen_._ Within the NANOG and SOX2 HREs, human ES cells maintained under atmospheric conditions exhibited high H3K9me3 levels, representing a marker of transcriptional silencing, and significantly reduced H3K4me3 and H3K36me3 compared to cells cultured under hypoxic conditions [116], consistent with a more closed conformational chromatin with atmospheric oxygen culture.

The oxygen concentration used to isolate human ES cells has been shown to impact X inactivation status, with those cultured under physiological oxygen able to maintain both active X chromosomes [117]. In contrast, those cultured under atmospheric conditions readily inactivate an X chromosome. SIRT activity is responsive to alterations in cellular redox including oxidative stress (reviewed by [118]) and may be one mechanism by which oxygen-regulated changes to the epigenome are established. Alternatively, HIF activation has been shown to upregulate MAT2A transcription in hepatoma cells, reducing SAM levels and leading to DNA demethylation [119]. Whether a similar relationship exists in pluripotent stem cells remains to be determined.

### 7.2. Regulation of Demethylation by Vitamin C

Vitamin C is commonly added to culture as an antioxidant, yet it has been shown to regulate DNA methylation dynamics in human and mouse pluripotent stem cells, acting as a key regulator of TETs [120] and the Jumonji family of histone demethylases [121]. The culture of human ES cells without added vitamin C increased DNA methylation, while the presence of vitamin C promotes DNA demethylation [122]. In mouse ES cells, supplementation with vitamin C leads to a rapid increase in 5hmC, dependent on Tet activity [123]. Addition of vitamin C to culture establishes an epigenetic landscape more similar to the inner cell mass of the embryo [123], potentially revealing a role for vitamin C in culture independent of its antioxidant capacity.

## 8. The Significance of Metaboloepigenetics to Pluripotent Stem Cell Biology

It is clear that metabolism can drive cell state transitions through interacting with the signalling machinery and more subtly through modification of the epigenome. What is less clear is how perturbations in metabolism impact subsequent potency and cell function. A consequence of perturbing metabolism is that heritable changes to DNA are passed on to daughter cells.

*In vitro* embryo culture has been found to be associated with alterations in DNA methylation and the expression of imprinted loci [124–128]. Assisted reproductive technologies that involve the culture of the preimplantation embryo have been associated with the early onset of metabolic disease (reviewed by [129]) and an increased frequency of epigenetic disorders [130] in offspring. Imprinting abnormalities following* in vitro* culture have been described in embryos cultured with different media formulations [131]. A key difference in these media formulations is the provision of amino acids, providing a correlation between metabolite concentration in the environment of the preimplantation embryo and lifelong impacts on the resulting child. The importance of establishing correct metabolic regulation in embryos in culture cannot be underestimated. There is still much work to do in medium optimization. Studies on the impact of the various commercially available media formulations on human ES cell epigenetics are essentially lacking.

Independently derived human ES cells display relatively stable methylation patterns [132] and share equivalent genomic arrangements [40]. These analyses do not encompass the entirety of the epigenetic landscape and smaller differences may exist between cell lines and between what is considered the norm of cells in culture versus cells in the embryo. A heavy reliance on glycolysis may, by default, activate the major pathways that regulate the epigenetic landscape, providing sufficient intermediates to enable the maintenance of pluripotency. However, subtle differences in medium formulation may impact less well-characterized modifications. The process of ES cell isolation must, by its very nature, place selective pressure on cells that is likely to be resolved, in part, through heritable modifications to the epigenome that embed changes in gene expression. Significant differences in the transcriptome of human ES cell and ICM cells have been shown, demonstrating that the process of ES cell derivation significantly alters gene expression [133] and providing evidence for this selective process. Adaptation may also involve modification to the metabolome of cells. ES cells, pluripotent by all standard measures, can display disparate metabolic profiles, suggesting metabolic adaptation can occur. It will be important to assess whether these changes in the metabolome impact, in turn, the pluripotent epigenome, eliciting changes in differentiation potential and/or cell function. These studies are not complete, and next-generation sequencing is required to establish a comprehensive characterization of the ES cell epigenome in culture to identify the impact of culture adaptation on epigenetic integrity.

### 8.1. The Transition from Differentiated to Pluripotent Cell State Is Accompanied by Changes in Metabolism

The introduction of pluripotency transcription factors to somatic cells brings about progressive loss of the somatic phenotype and the acquisition of a pluripotent-like cell state (induced pluripotent stem; iPS cells). Reprogramming to a pluripotent-like state requires the remodelling of both metabolism (reviewed by [9]) and chromatin organization (reviewed by [134]). Genome-wide chromatin remodeling is initiated in response to reprogramming factor expression [135] establishing an epigenetic profile similar to that of embryonic stem cells, where key developmental genes remain poised in a bivalent (repressed but activatable) state [136, 137]. Acquisition of a pluripotent-like state necessitates the upregulation of glycolysis and downregulation of oxidative phosphorylation [77, 78, 138]. Appropriately modulating metabolism is essential to establish the pluripotent cell state, evidenced by the ability to enhance or reduce reprogramming efficiency through metabolite modulation, including through modulation of oxygen concentrations [77, 139, 140]. Despite the capacity to acquire a number of pluripotent characteristics, iPS cells are not physiologically equivalent to their ES cell counterparts.

Differences in metabolism between ES and iPS cells have been described. Increased levels of SAM pathway metabolites and differences in unsaturated fatty acids are seen in iPS cells [141], suggesting that the reprogramming of metabolism is incomplete or perturbed in these cells. We have documented alterations in the capacity of iPS cells to regulate metabolism in response to oxygen (Harvey et al, unpublished). iPS cells have been shown to retain an epigenetic memory of their somatic origin [142–145], which is perpetuated through differentiation [144–146]. Conceivably, the higher level of global DNA methylation in iPS cells is evidence of the inappropriate regulation of metabolism during reprogramming. Several factors used to reprogram somatic cells are known regulators of metabolism. For example, Lin28a has been shown to enhance the translation of mRNAs for several metabolic enzymes and thereby to regulate glycolysis and OXPHOS [147]. These findings raise questions on the potential roles these factors will play, if any, in modulating pluripotent stem cell metabolism and how their impact on iPS cell metabolism is reflected in the epigenome. Epigenetic modifications could conceivably impact the physiology of iPS disease models or the utility of these cells in drug discovery or regeneration.

## 9. Conclusions

Many factors impact the relative activity of metabolic pathways and the composition of metabolite pools within the cell, including the extracellular milieu, the regulation of the cell: environment interface, cell identity and function, and the stress imposed on the cell by extracellular and intracellular regulators. Metabolites, such as acetyl-CoA and SAM, connect metabolism to signaling and gene expression. The availability of these compounds also impacts epigenetic modifications in the cell, with low levels resulting in reductions in acetylation and methylation, respectively. Evidence, as reviewed here, suggests that the metabolome plays a defining role in the epigenetic regulation of the cell, including cells of the pluripotent lineage. What is equally clear from the available evidence is that there is much more work needed to describe the role of metabolism in the epigenome and then to understand the biological programs regulated by metabolically controlled epigenetic mechanisms. Integration of how metabolism changes with cell states is needed, as the majority of studies to date fail to delineate between cell states or, more specifically, address the transitions between them.

In culture, the ability of a cell to* adapt* to its environment may be reflected in changes to the epigenome. These changes are selected to promote survival in an environment defined by nutrient availability and effected through the activity of metabolic pathways and are* perpetuated* within the cell population. Despite this knowledge, the impact of different media used for pluripotent stem cell maintenance and iPS cell generation is poorly appreciated, where the quality of cells within a medium is generally evaluated without metabolic analysis. As with the blastocyst/inner cell mass, an inappropriate nutrient composition for the culture of pluripotent cells may compromise “metabolic fidelity” and have significant downstream impacts on development and viability. It is likely that these impacts are mediated through epigenetic regulation, and, in culture, alterations will be perpetuated with cell division. Selected changes in metabolism could limit the availability of cofactors, like SAM and acetyl-CoA with long term, heritable alterations to the epigenome. These could impact the identity of daughter cells, bias differentiation potential, or compromise the function of differentiated derivatives, potentially in subtle but important ways.

Elucidating the dynamics of, and mechanisms that control, cellular responses to metabolite availability will provide opportunities to manipulate cell fate. Establishing the appropriate balance of nutrients to support ongoing development requires a clearer understanding of the regulation of the pathways modulating metabolic control and identification of mechanisms that are perturbed by specific environmental conditions. Future studies should address the impact of metabolic adaptations of pluripotent stem cells to various culture conditions in the absence of changes to the base formulation, the metabolic regulation of differentiation, and how differences in metabolism impact cell function. As epigenetic landscapes can impact disease states, including cancer and neurodegenerative disorders, the appropriate regulation of these enzymes established through metabolic pathways will rely on the establishment of physiologically relevant conditions to support the continuum of pluripotent stem cell states. The impact of culture protocols on downstream epigenetic profiles and differentiated cell function will need to be investigated to inform of any deleterious conditions that negatively alter cell physiology.

## Figures and Tables

**Table 1:** Summary of metabolites linked with epigenetic modifications in pluripotent stem cells.

Metabolite	Epigenetic intermediate	Metabolic pathway	Epigenetic target	References
Threonine	SAM (via glycine)	Folate/SAM cycles	H3K4me3	[60–62]
	Acetyl-CoA			

Methionine	SAM	SAM cycle	H3K4me3	[64]

Glutamine	*α*KG	TCA	H3K9me3	[65]
			H3K27me3	
			H3K26m3	
			H4K20me3	

l-Proline	*TBD*	*TBD*	MRT	[74]
			H3K9me	
			H3K36me	

Glucose	Acetyl-CoA	(glycolysis-derived)	H3K9/K27ac	[76]
	(from citrate)	Pyruvate oxidation		

*TBD*	*TBD*	O-GlcNAc	me/TETs	[83]

*TBD*	NAD^+^	*TBD*	ac/sirtuins	—

## Abbreviations

ac: Acetylation
ACL: ATP citrate lyase
*α*KG: Alpha-ketoglutarate
ATP: Adenosine triphosphate
F-6-P: Fructose-6-phosphate
G-6-P: Glucose-6-phosphate
G6PD: Glucose-6-phosphate dehydrogenase
Glu: Glutamate
GSH: Glutathione
HATs: Histone acetyltransferases
HMT: Histone methyltransferases
HCY: Homocysteine
JMJ: Jumonji demethylases
5mC: Methylcytosine
5hmC: Hydroxymethylcytosine
LDH: Lactate dehydrogenase
me: Methylation
Met: Methionine
NAD^+^: Nicotinamide adenine dinucleotide
NADH: Reduced form of NAD^+^
NADP^+^: Nicotinamide adenine dinucleotide phosphate
NADPH: Reduced form of NADP^+^
O_2_: Oxygen
O-GlcNAc: O-linked *N*-acetyl glycosylation
OXPHOS: Oxidative phosphorylation
PDH: Pyruvate dehydrogenase
PKM2: Pyruvate kinase M2
PPP: Pentose phosphate pathway
Pro: Proline
SAH: S-Adenosylhomocysteine
SAM: S-Adenosyl methionine
SIRTs: Sirtuins
TCA: Tricarboxylic acid cycle
TET: Ten-Eleven Translocation demethylases
Tdh: Threonine dehydrogenase
THF: Tetrahydrofolate
5-CH_3_-THF: 5-Methyltetrahydrofolate
Thr: Threonine.
